# An unexpected finding from a submental mass

**DOI:** 10.1259/bjrcr.20180094

**Published:** 2019-01-25

**Authors:** Meeral Makwana, Stephen Walsh, Sameer Gangoli

**Affiliations:** 1 Western Sussex Hospitals NHS Trust, Chichester, UK

## Abstract

This case describes a fit and well 17-year-old male who underwent surgical resection of a longstanding, painless, right lateral neck swelling. Believed to be either a vascular malformation, ranula or enlarged sublingual gland from pre-operative MR studies, histopathological examination of the mass revealed it as normal thyroid tissue. Post-operative imaging confirmed the absence of any remaining thyroid tissue. Hypothyroidism was confirmed with subsequent thyroid function tests. Interestingly, a “thyroid storm” which presented unknowingly during the surgical removal of the lesion did not trigger suspicion that thyroid tissue was being handled at the time. Normal, ectopic thyroid tissue in the lateral neck is rare but should be considered a differential diagnosis for neck lumps, particularly if it also presents as an intraoral swelling, as in this case. The presence of the orthotopic thyroid gland should be confirmed with diagnostic imaging prior to surgical excision of unknown neck masse

## CASE PRESENTATION

**Figure 1.**
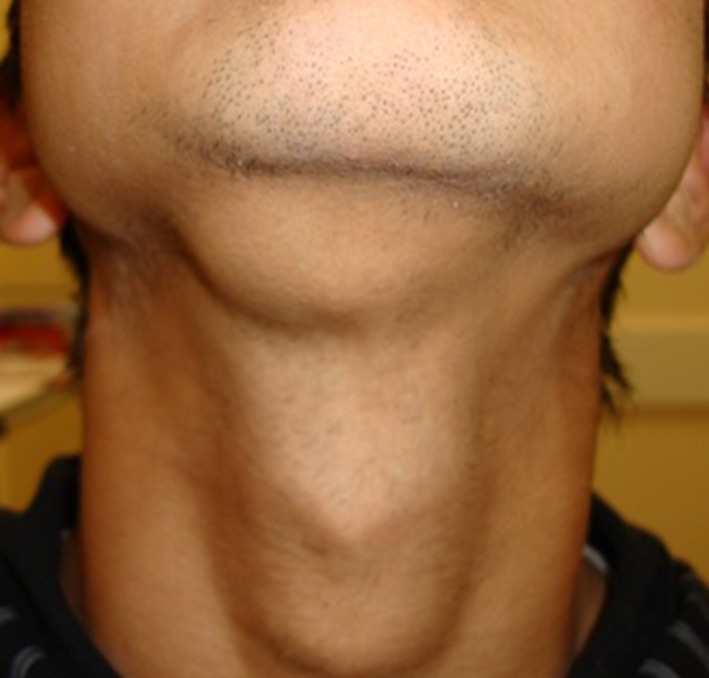
Asymmetric swelling in the submental region.

A fit and well 17-year-old Nepalese male was referred by his general medical practitioner (GMP) to the oral and maxillofacial unit for assessment of a large lump under his chin (Figure 1). The painless, slow growing mass has remained static in site for over 4 years. It did not move with swallowing or fluctuate with mealtimes, nor was it associated with any discharge extra or intraorally. There were no systemic features and the patient’s main concern was its appearance. The medical history was unremarkable, and he was a non-smoker.

Clinically a 3 × 4 cm, well-circumscribed, firm and non-fixed mass was present in the right submental region. It was indistinguishable from the submandibular gland and palpable bimanually, appearing to be contiguous with the adjacent mucosa intraorally. The lesion did not blanch under digital pressure and normal clear expression of saliva was observed from the submandibular glands.

NBimage has been coned in to improve anonymisation of the patient.

## INVESTIGATIONS & DIFFERENTIAL DIAGNOSES

The clinical impression was that this mass was not dentoalveolar in its origin. Flexible naso-endoscopy and a dental orthopantomogram film were unremarkable. Initial differential diagnoses included:

- A plunging ranula arising from the sublingual gland
- A plunging ranula with previous subclinical infection (suspected fibrosis giving rise to the firm texture)
- A benign enlargement of the right sublingual gland
- Pathology or tumour within the right sublingual gland
- A systemic manifestation of tuberculosis

**Figure 2.**
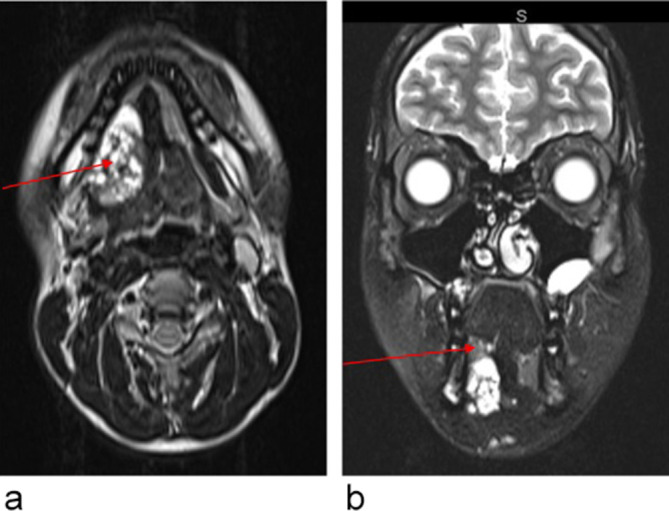
(a) (Axial *T*_2_). Arrow showing the mass within right sublingual space, *T*_2_ hyperintense. (b) (Coronal *T*_2_ fat sat) (c) and 2d removed. Arrow showing the right sublingual gland.

**Figure 3.**
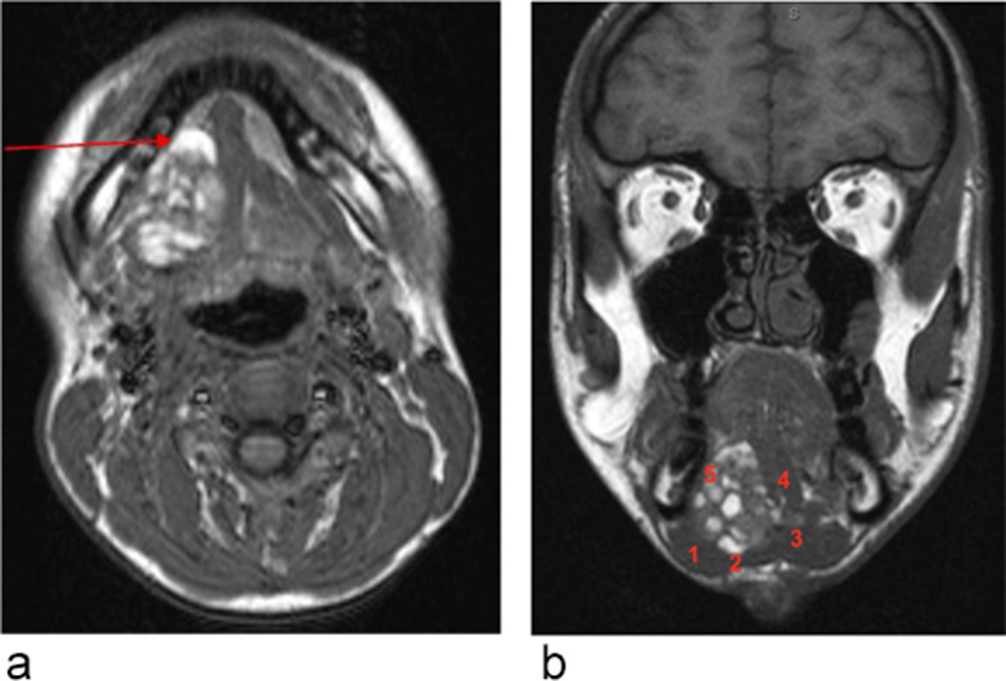
(a) (Axial *T*_1_). Arrow showing the intact lingual cortex of mandible adjacent to the hyperintense mass. (b) (Coronal T1). (1) Anterior belly of digastric muscles; (2) Mylohyoid; (3) Geniohyoid; (4) Genioglossus; (5) The unknown mass.

An MRI study of the head and neck was performed to evaluate the lesion further. It demonstrated a moderate sized mass (3.5 × 2.5 × 3.4 cm) within the right sublingual space which did not appear to breach the mylohyoid. It was well-defined with multiple thin-walled cystic spaces which demonstrated both *T*_2_ (Figure 2a–b) and *T*_1_ hyperintense (Figure 3a–b) signal return, suggestive of fluid and proteinaceous or methaemoglobin content.

**Figure 4.**
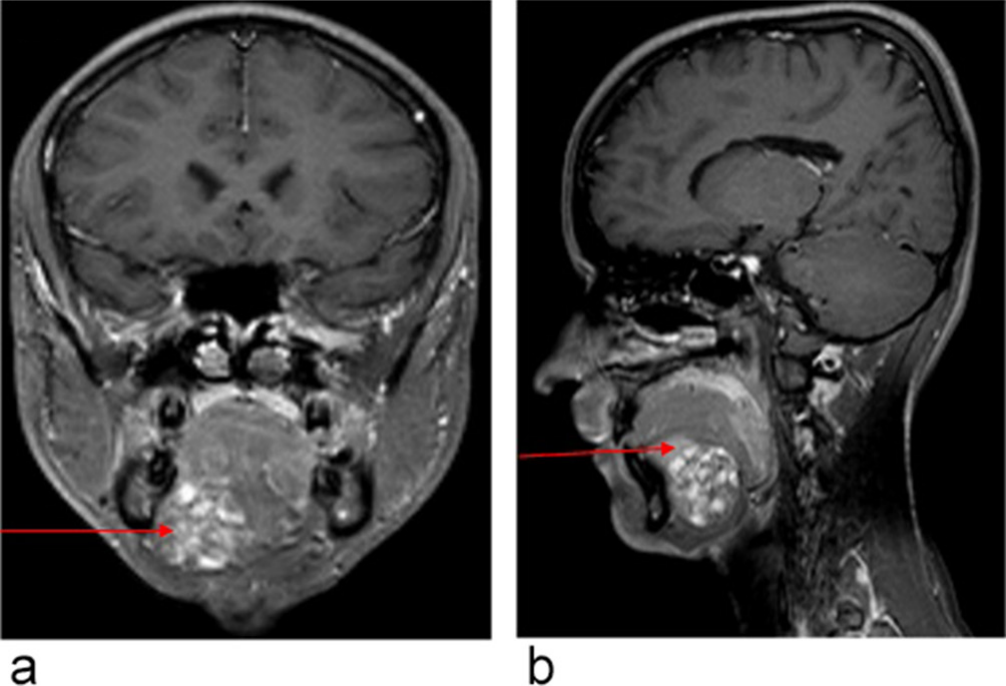
(Post-contrast axial *T*_1_ fat sat). Arrow demonstrating the mass with no appreciable contrast enhancement. (b) (Post-contrast coronal *T*_1_ fat sat). Arrow showing the right sublingual gland, intact and above the unknown mass.

There was no appreciable contrast enhancement (Figure 4a–b), but assessment was limited due to the T1 hyperintensity on the non-contrast images. The lesion crossed the midline, but there was no extension into the submandibular space and there was no clear invasion of the mandible (Figure 3a) or of the tongue base (Figure 3b).

The rest of the salivary glands were normal, as were the pharyngeal and laryngeal structures and there was no cervical adenopathy. The most likely diagnosis suggested by the reporting radiologist was a cavernous/cystic lymphangioma in the right sublingual space, with differential diagnoses including a vascular malformation or dermoid cyst.

Further assessment by a head and neck/maxillofacial radiologist was advised. The latter review again described the lesion as displacing the right sublingual gland superiorly and the right mylohyoid inferiorly and abutting the lingual plate of the mandible with no evidence of cortical breach. They also suggested a cavernous or cystic lymphangioma, or a complex cystic ranula of a minor salivary gland.

These possibilities are described in Table 1 below, with the lymphangioma appearing most likely due to the imaging features.

**Table 1.** Differential diagnoses following the MR study

	Features in favour	Features against
Lymphangioma	Multicystic *T*_1_ hyperintense Lack of enhancement	
Ranula (minor salivary gland)	Position	Complex with *T*_1_ hyperintensities
Haemangioma	Multicystic *T*_1_ hyperintense	Lack of enhancement
Dermoid	Multicystic *T*_1_ hyperintense Lack of enhancement	Eccentric position
Paraganglioma	Dark foci within mass on *T*_2_ weighted images appearing as flow voids	Lack of classic flow voids (similar foci not evident on *T*_1_ weighted images) Lack of classic “salt and pepper” appearance
Cystic schwannoma	Multicystic *T*_1_ hyperintense	Lack of enhancement
Minor salivary gland tumour	Heterogenous mass with cystic areas	Lack of enhancement
Tuberculosis/ other rare infections? Actinomycosis	Heterogenous mass with cystic areas	No clinical suspicion No other adenopathy

Histological correlation of the lesion was advised. However, biopsy or fine needle aspiration were not performed due to the risk of haemorrhage from this suspected vascular lesion.

## TREATMENT

Excisional biopsy was advised to address the patient’s concern and to acquire a histological diagnosis. Other advantages of surgery included prevention of continued enlargement of the mass and potential encroachment on adjacent structures. Specific risks included paraesthesia to the right lower lip, tongue and chin, need for the mandibulotomy and loss of anterior teeth to allow adequate access, neck scarring and salivary fistula, in addition to pain, bleeding, bruising, swelling and infection.

The mass was surgically removed under general anaesthetic using a trans-oral approach. The right submandibular gland and lingual nerve were identified and protected and the lesion was dissected and delivered whole. Intraoperatively, the patient became inexplicably hypertensive and tachycardic on manipulation of the mass. This was resistant to medical management but recovered spontaneously. The patient was tachycardic (129–143 beats per minute) for 30 h following surgery, at which point all observations had normalised and the patient was discharged. This was retrospectively diagnosed as acute thyrotoxicosis or a “thyroid storm”.

## OUTCOME AND FOLLOW-UP

The patient made an excellent recovery and denied any facial numbness at a 2-week review. In stark contrast to the provisional diagnoses, the histopathological report reported that the excised mass was normal thyroid tissue. The entire specimen was consistent with lingual thyroid tissue with no atypical or pathological features.

*“A nodular area of normal thyroid tissue comprising small and large colloid filled follicles with scattered haemosiderin laden macrophages suggestive of previous haemorrhage.* *”*

Urgent thyroid function tests and calcium checks were performed. These showed deranged levels of thyroid function: TSH > 100 [normal serum TSH level 0.1–5 mu l^–^^1^] and free T4 3.7 [normal serum free T4 level 9–21 pmol l^−1^] confirming hypothyroidism.

**Figure 5.**
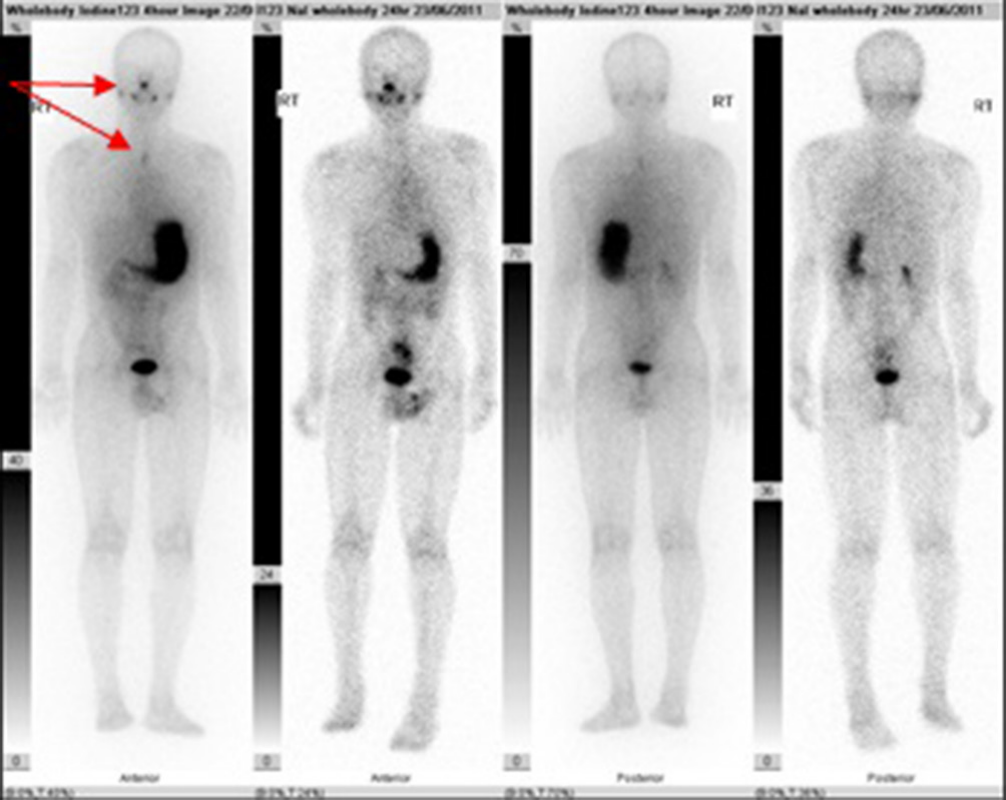
Post-operative iodine uptake. Arrows showing physiological uptake in the nasal mucosa and salivary glands and upper oesophagus.

**Figure 6.**
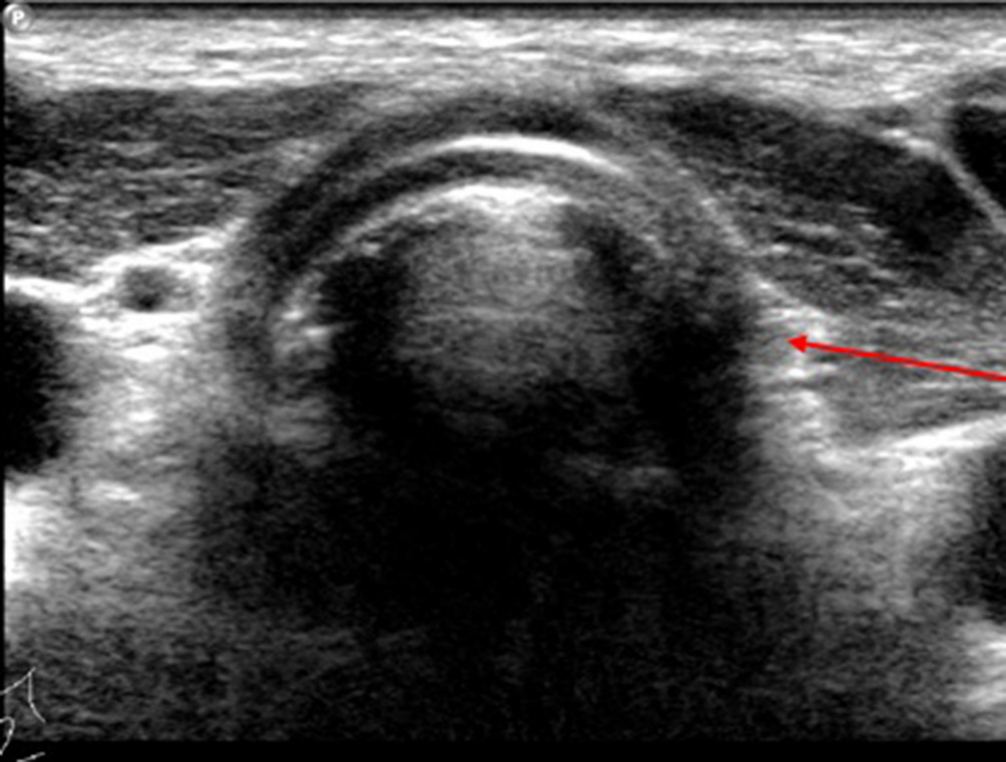
Post-operative neck ultrasound demonstrating trachea and strap muscles. The brightly reflective bow tie structure anterior to the trachea representative of normal thyroid is absent.

Due to the histology obtained and noting that the initial imaging studies did not include the thyroid region, the patient had post-operative radionuclide scan with I-131 (Figure 5) and ultrasound (Figure 6) studies confirming the lack of normal thyroid tissue in the expected location.

The arrow shows the expected position of the left lobe of the thyroid gland.

The patient was commenced on 150 micrograms of thyroxine, continued by the GMP. TSH and T4 levels continued to be monitored thereafter. However, the GMP grew concerned by the patient’s poor compliance to the medication after 2 months. The patient also failed to attend multiple follow-up appointments at the oral and maxillofacial surgery department. He was contacted in writing where it was impressed upon him the importance of continuing the medication and the need for life-long monitoring. Fortunately at a further clinic review, the patient’s compliance had improved and thyroid function tests were restored to normal levels (TSH 0.5 and free T4 19.4).

## DISCUSSION

The thyroid gland assumes its normal position 7 weeks post-fertilisation below the larynx and hyoid bone, anterolateral to the second, third and fourth tracheal cartilaginous rings.^1,2^ Development begins at the pharyngeal floor and the gland begins to descend at 4 weeks as a diverticulum from an invagination at the foramen caecum. It remains connected to the tongue via the thyroglossal duct which solidifies and is normally obliterated by the eighth week of gestation .^1,2^

Disturbances during embryogenesis can lead to an abnormal development of the gland resulting in anomalous locations of the thyroid tissue. Ectopic thyroid tissue is defined as “thyroid tissue not located anterolaterally to the second and fourth tracheal cartilages.”^3,4^ Most ectopic presentations are found at the base of the tongue and in the midline whilst lateral aberrant presentations are extremely rare.^2^ Approximately 1 to 3% of all ectopic thyroids are located in the lateral neck, of which 70% are submandibular.^1^

When thyroid tissue is present in an ectopic location along with a eutopic thyroid, it is referred to as “accessory thyroid,” but the existence of ectopic thyroid glands at two different locations is very rare.^4^ True ectopic thyroid is when thyroid tissue is absent in the normal location as demonstrated in this case. While an absent normal thyroid can make diagnosis easy, accessory thyroid tissue in addition to normal thyroid can complicate diagnosis.

Prado’s algorithm (2012) advises ultrasound early on during the diagnostic work up for a suspected ectopic thyroid. Ultrasound scan should follow clinical palpation for the orthotopic thyroid gland but precede CT or MRI, fine needle aspiration biopsies and thyroid function testing.^1^ An ultrasound scan is a non-invasive, simple and cost-effective study which was not performed at baseline in this case. It offers no exposure to ionising radiation unlike scintigraphy, CT or MRI, and with the use of the colour Doppler technique, sensitivity in detecting ectopic thyroid tissue is increased.^4,5^

Any palpable lesion within the neck should be evaluated with a complete high-resolution neck ultrasound first which includes assessment of all the salivary and thyroid glands as well as the lymph nodes. The absence of a normally located thyroid gland would have influenced the differential diagnoses from the outset and prevented unnecessary iatrogenic hypothyroidism.

An advantage that a CT scan may have over MRI is the display of uniform high attenuation, similar to a normal thyroid gland, which is characteristic of ectopic thyroid tissue.^4^ Ibrahim and Fadeyebi (2011) report that MRI can show an elevated signal on *T*_1_ and *T*_2_ weighted images compared with the surrounding musculature and is more useful for lingual thyroid identification particularly when there is difficulty in differentiating thyroid tissue from tongue muscle. Further advantages of CT include a lower cost of the procedure and shorter imaging time whereas MRI offers less radiation exposure than CT.

Ectopic thyroid tissue should be considered as a differential diagnosis for lateral neck masses alongside submandibular gland pathology and tumours, inflammatory lesions (*i.e.* Küttner tumour), branchial cleft cysts, lymphangiomas, carotid body tumors and lymphadenopathy.^1–3^ Absent suspicion of an ectopic thyroid gland in this case meant that the tachycardia and hypertension observed during surgical removal of the neck mass were not attributed to a thyroid storm at the time of surgery. However, on reflection, this phenomenon was likely to have been an episode of acute thyrotoxicosis, precipitated by physical manipulation of the mass, leading to sudden release of T3 and T4 thyroid hormones into the bloodstream.^6^

This case report highlights the need to always document the presence and the location of the thyroid gland, which is becoming fast forgotten in this day and age of limited focused ultrasound imaging.

## LEARNING POINTS

1. Ectopic thyroid tissue should be considered as a differential diagnosis for lateral neck masses.
2. A neck swelling contiguous with intraoral swelling should not deter the suspicion of an ectopic thyroid (although thyroid tissue above mylohyoid is rare) and requires a baseline complete ultrasound examination of the neck including the thyroid region.
3. Ultrasonography, ^99m^Tc or iodine-131 scintigraphy, CT/MRI, Fine-needle aspiration biopsy and Thyroid function testing are essential in the workup of an ectopic thyroid.
4. If ectopic thyroid is not suspected and/ or an ultrasound neck not ordered, the CT or MRI examination should not fail to report the presence or absence of a normal thyroid gland in the evaluation of a neck mass.
5. A CT study may have an advantage over MRI by displaying a hyperdense gland on plain scans (characteristic uniform high attenuation similar to a normal thyroid gland) with intense post-contrast enhancement.
6. Optimal evaluation of a neck mass and suspicion of ectopic thyroid is necessary to prevent unnecessary surgical intervention and iatrogenic hypothyroidism that has long-term consequences.
